# Potential of spectroscopic analyses for non-destructive estimation of tea quality-related metabolites in fresh new leaves

**DOI:** 10.1038/s41598-021-83847-0

**Published:** 2021-02-18

**Authors:** Hiroto Yamashita, Rei Sonobe, Yuhei Hirono, Akio Morita, Takashi Ikka

**Affiliations:** 1 Faculty of Agriculture, Shizuoka University, 836 Ohya, Suruga-ku, Shizuoka, 422-8529 Japan; 2 United Graduate School of Agricultural Science, Gifu University, 1-1 Yanagito, Gifu, 501-1193 Japan; 3 Institute for Tea Science, Shizuoka University, 836 Ohya, Suruga-ku, Shizuoka, 422-8529 Japan; 4 Division of Tea Research, Institute of Fruit Tree and Tea Science, National Agriculture and Food Research Organization (NARO), 2769 Shishidoi, Kanaya, Shimada, Shizuoka 428-8501 Japan

**Keywords:** Biochemistry, Biological techniques, Computational biology and bioinformatics, Plant sciences

## Abstract

Spectroscopic sensing provides physical and chemical information in a non-destructive and rapid manner. To develop non-destructive estimation methods of tea quality-related metabolites in fresh leaves, we estimated the contents of free amino acids, catechins, and caffeine in fresh tea leaves using visible to short-wave infrared hyperspectral reflectance data and machine learning algorithms. We acquired these data from approximately 200 new leaves with various status and then constructed the regression model in the combination of six spectral patterns with pre-processing and five algorithms. In most phenotypes, the combination of de-trending pre-processing and Cubist algorithms was robustly selected as the best combination in each round over 100 repetitions that were evaluated based on the ratio of performance to deviation (RPD) values. The mean RPD values were ranged from 1.1 to 2.7 and most of them were above the acceptable or accurate threshold (RPD = 1.4 or 2.0, respectively). Data-based sensitivity analysis identified the important hyperspectral regions around 1500 and 2000 nm. Present spectroscopic approaches indicate that most tea quality-related metabolites can be estimated non-destructively, and pre-processing techniques help to improve its accuracy.

## Introduction

Plants collectively produce many metabolites with estimates ranging from 100,000 to 1 million, and many metabolites are thought to play essential roles in resistance to biotic stresses and tolerance of abiotic stresses^1–5^. In addition, natural products synthesized in plants provide indispensable resources for human health and survival^5^. Given the importance of plant metabolites to plant development and adaptation, and for human health, various quantitative and qualitative analyses have been developed. The main examples are based on chromatography techniques such as gas chromatography or high-performance liquid chromatography (HPLC) with improved mass resolution and sensitivity^6,7^. However, these analytical methods require the destructive collection and pre-treatment of plant samples, which makes them slow in acquiring analytical data and unsuitable for real-time diagnosis of metabolite level.

Hyperspectral reflectance sensing is an established spectroscopic method that can provide rapid analysis without the need for sample pre-treatment. It is commonly applied to visible (VIS; 400–700 nm), near-infrared (NIR; 700–1000 nm), and short-wave infrared (SWIR; 1000–2500 nm) spectral ranges and has been used to estimate leaf pigments and water contents^8,9^. The VIS is dominated by absorption of the photosynthetic pigments such as chlorophylls, carotenoids, and anthocyanins^8^. On the other hand, NIR spectroscopy is directly relevant to the overtones and combinations of the fundamental C–H, O–H, and N–H bonds in organic molecules^10,11^. Thus, NIR spectroscopy provides physical and chemical information and has shown good potential in estimating different parameters in biotic samples, including metabolites in plants, agricultural products, and food^12–14^. In addition, machine learning techniques provide powerful tools for constructing regression or classification models in agricultural indices from hyperspectral reflectance data^15^. The methodology of machine learning algorithms provides a flexible model not only for data-driven decision-making but also for capturing expertise into the algorithms^16^. The technique shows good potential for analyzing hyperspectral reflectance data with all spectral information based on a large number of bands^17^. Machine learning techniques also enable the assessment of hyperspectral features that are informative for high accuracy predictive modelling^16,18^.

Tea plants (*Camellia sinensis* L.) are mainly distributed and cultivated in Asia to produce several tea types, such as green tea, oolong tea, and black tea, which are popular non-alcoholic beverages consumed all over the world. Tea-drinking reportedly has numerous and diverse health benefits^19^. Generally, tea quality and function are defined by the profile of various chemical components, such as catechins, caffeine, and theanine, which are characteristics to tea leaves. Tea catechins, which comprise a major class of polyphenols, contribute to the taste of astringency and bitterness of tea and have been studied for their health functions such as antibacterial activities^20^ and free radical scavenging activities^21^. Free amino acids, especially glutamate (Glu) and theanine, contribute to the *umami* taste of green tea^22,23^. In particular, theanine, a unique amino acid in tea plants, has the activities of promoting relaxation^24^ and reducing blood pressure^25^. Caffeine (1,3,7-trimethylxanthine) is a kind of purine alkaloid and its consumption may be associated with a reduced risk for type 2 diabetes^26^, but excessive intake of caffeine may cause inflammation of the digestive organs, insomnia, and arrhythmia^27^. Thus, unique tea quality-related metabolites are the most important agronomic traits targeted by modern and future tea cultivation and breeding. To evaluate the levels of these metabolites, many analytical tools have been employed to quantify tea quality-related metabolites including free amino acids, catechins, and caffeine contents in tea samples. Many analytical methods have been based on HPLC^28,29^ and capillary electrophoresis^30,31^, but these methods destructively use plant tissues and are time-consuming and expensive to perform. Therefore, a rapid and accurate method for the evaluation of quantitative traits in tea leaves is in high demand for tea cultivation management and breeding programs. The NIR-based estimation of some chemical components in ground tea leaves has been established by previous studies^32–34^. Few studies have been reported in a non-destructive method for fresh leaves^35,36^. Huang et al.^35^ have reported non-destructive estimation methods for four main catechins and caffeine in fresh green leaves based on VIS–NIR spectra (400–2498 nm) and partial least squares (PLS) model. However, the outcomes of this study were limited by fewer tea quality-related metabolites and the sample status from leaf positions and fewer tea quality-related metabolites, which cannot achieve robust results in actual agricultural management.

We have achieved the non-destructive estimation of chlorophyll and nitrogen contents in tea leaves by combining the VIS–NIR–SWIR (400–2500 nm) hyperspectral reflectance data and machine learning algorithms^37^. In the current study, we acquired the reflectance and 15 tea quality-related metabolites traits from the various nitrogen conditions, the leaf-stage, shading conditions, and albino tea leaves to construct the robust models. Pre-processing techniques and machine learning algorithms for hyperspectral data were used to perform regression modelling to non-destructively estimate the contents of free amino acids, catechins, and caffeine as tea quality-related metabolites in new fresh leaves. Our modelling indicated that most tea quality-related metabolites can be estimated by VIS–NIR–SWIR hyperspectral reflectance data and machine learning algorithms and that pre-processing techniques help to improve its accuracy. In particular, the combination of de-trending (DT) pre-processing methods and Cubist algorithms showed the highest model performance for most tea quality-related metabolites.

## Results

### Data distribution of reflectance data and tea quality-related metabolite contents

**Figure 1:**
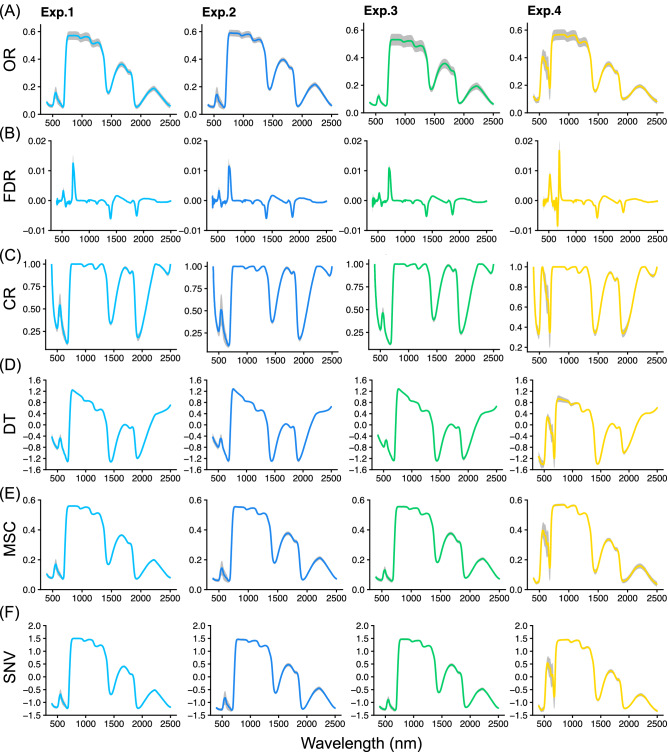
Pre-processing spectral patterns of original reflectance (OR) in tea leaves. Five pre-processing techniques were applied to the OR (**A**) base: first derivative reflectance (FDR, **B**), continuum-removed (CR, **C**), de-trending (DT, **D**), multiplicative scatter correction (MSC, **E**), and standard normal variate transformation (SNV, **F**). Colors in spectra (Exp. 1, light blue; Exp. 2, blue; Exp. 3, green; Exp. 4, yellow) and gray indicate mean and standard deviation, respectively. Figures were visualized by the R package “ggplot2” ver. 3.3.2.

**Figure 2:**
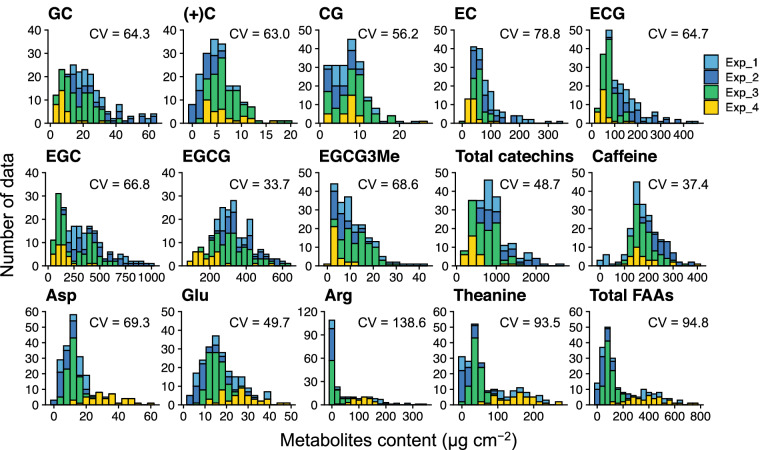
Data distribution of 15 phenotypes for tea quality-related metabolites. Number of samples: 201, 201, and 215 for catechins, caffeine and free amino acids (FAA), respectively. Coefficient of variation (CV) value for each metabolite is included on the Figure. Figures were visualized by the R package “ggplot2” ver. 3.3.2.

Original reflectance (OR) data were obtained at 1-nm steps across the 400 to 2500 nm wavelength from approximately 200 leaves in four experiment conditions. Five pre-processing methods, namely first derivative reflectance (FDR), continuum-removed (CR), standard normal variate (SNV), multiplicative scatter correction (MSC), and DT, were applied to the OR data. Several spectral patterns were observed in OR and pre-processed reflectance (Fig. 1). In the same leaves that were measured by reflectance, we analyzed catechins, caffeine, and FAAs as tea quality-related metabolites by HPLC and acquired 15 phenotypic traits. For catechins, the contents of (+)-gallocatechin (GC), (+)-catechin (C), (−)-epicatechin (EC), (−)-epigallocatechin (EGC), (−)-catechin gallate (CG), (−)-epicatechin gallate (ECG), (−)-epigallocatechin gallate (EGCG), (−)-epigallocatechin-3-*O*-(3-*O*-methyl)-gallate (EGCG-3ʺMe), and total catechins were in the ranges of 3.4–64.6, 0.5–19.2, 1.1–25.3, 8.4–339.4, 21.4–459.4, 46.8–1003.1, 91.0–619.8, 1.3–43.3, and 206.2–2528.7 μg cm^−2^, respectively (Fig. 2). For FAAs, the contents of aspartate (Asp), glutamate (Glu), arginine (Arg), theanine (Thea), and total FAAs were in the ranges of 1.6–59.3, 3.1–49.1, 0.9–346.4, 0.2–264.5, and 12.3–746.0 μg cm^−2^, respectively (Fig. 2). Caffeine content was in the range of 1.8–393.1 μg cm^−2^ (Fig. 2). The coefficient of variation (CV) in 15 phenotypes was in the range of 33.7%–138.6% (Fig. 2).

### Best combination of pre-processing and machine learning algorithms in regression model performance

**Figure 3:**
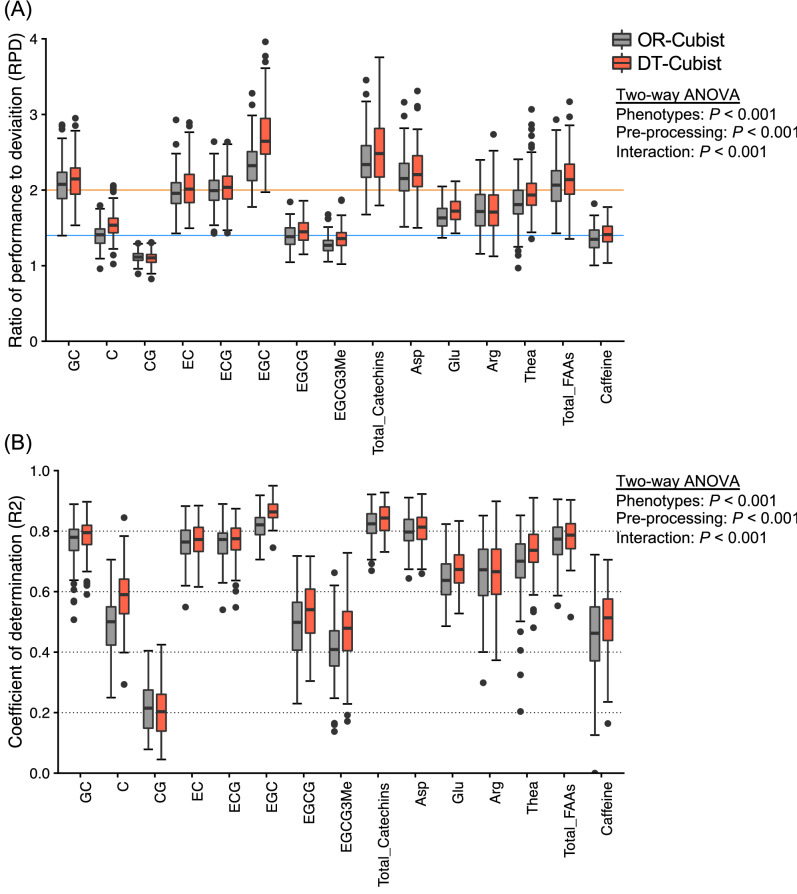
Model performance and robustness based on OR-Cubist and DT-Cubist for tea quality-related metabolites. The ratio of performance to deviation (RPD, **A**) and coefficient of determination (R^2^, **B**) were applied to evaluate the accuracy of each model. A stratified sampling approach for modelling was repeated 100 times to obtain robust results. Figure are plots of the RPD and R^2^ values in each repeat. Orange and blue lines indicate RPD values of 1.4 and 2.0, respectively, as accuracy thresholds. Statistical tests for significant differences by two-way ANOVA are shown on the right side of the Figure. Figures were visualized by the R package “ggplot2” ver. 3.3.2.

Using six spectral patterns (OR, FDR, CR, SNV, MSC, and DT) and five machine learning algorithms, Random Forest (RF), Support Vector Machine (SVM), Cubist, Stochastic Gradient Boosting (SGB), and Kernel-based Extreme Learning Machine (KELM), we performed regression modelling for 15 phenotypes of tea quality-related metabolites (Supplementary Fig. S1). Model performances in the combination of pre-processing and machine learning algorithms were evaluated based on the ratio of performance to deviation (RPD) values and robustness over 100 repetitions (Supplementary Table S2). In most phenotypes, the combination of DT and Cubist (DT-Cubist) was selected most often as the best performing combination in each round among the 100 repetitions (Table 1, Supplementary Table S2). The model performance based on DT-Cubist was different between the 15 phenotypes (Fig. 3A; two-way ANOVA, *P* < 0.001). Except for CG and EGCG-3ʺMe, the mean RPD values in most of them were above the acceptable threshold (RPD = 1.4)^38^. In GC, EC, ECG, EGC, total catechins, Asp, and total FAAs, the mean RPD values were above the accurate threshold (RPD = 2.0)^38^. The modelling based on DT-Cubist significantly increased model performance over that based on OR-Cubist (Fig. 3A; two-way ANOVA, *P* < 0.001). These results were also supported by the root-mean-square error (RMSE) values and the coefficient of determination (R^2^) values as a model performance index (Fig. 3B, Table 2).

**Table 1:** Best combination of pre-processing and machine learning algorithms after 100 repetitions.

Phenotypes		Most selected pre-processing and models	Frequency (100 repeat^−1^)
Catechins	GC	DT-cubist	32
	C	SNV-cubist	17
	CG	DT-RF, OR-cubist	8
	EC	DT-cubist	18
	ECG	DT-cubist	23
	EGC	DT-cubist	65
	EGCG	DT-cubist	37
	EGCG3Me	DT-cubist	23
	Total	DT-cubist	42
FAAs	Asp	DT-cubist	23
	Glu	DT-cubist	50
	Arg	DT-cubist	25
	Thea	DT-cubist	27
	Total	DT-cubist	34
Caffeine		DT-cubist	20

Combination of pre-processing and machine learning algorithms were evaluated based on RPD values.

**Table 2:** Summary of validation and prediction performance based on DT-Cubist in 15 phenotypes for tea quality-related metabolites.

Phenotypes		Observed values (mg cm^−2^)			DT-cubist																		Model performance^a^
					RPD*v*			R^2^*v*			RMSE*v* (mg cm^−2^)			RPD*p*			R^2^*p*			RMSE*p* (mg cm^−2^)			
Catechins	GC	21.2	±	13.7	2.22	±	0.30	0.79	±	0.06	5.96	±	1.01	2.10	±	0.30	0.78	±	0.06	6.70	±	0.90	Accurate
	C	5.3	±	3.3	1.56	±	0.20	0.59	±	0.11	2.20	±	0.43	1.50	±	0.20	0.58	±	0.10	2.10	±	0.30	Acceptable
	CG	7.5	±	4.2	1.18	±	0.11	0.29	±	0.12	3.56	±	0.56	1.10	±	0.10	0.21	±	0.09	3.80	±	0.50	Poor
	EC	67.4	±	53.2	2.23	±	0.38	0.80	±	0.07	23.86	±	4.80	2.00	±	0.30	0.77	±	0.06	27.70	±	4.70	Accurate
	ECG	119.3	±	77.2	2.17	±	0.29	0.79	±	0.06	33.66	±	5.82	2.00	±	0.20	0.77	±	0.06	40.00	±	5.30	Accurate
	EGC	317.6	±	212.1	2.80	±	0.37	0.87	±	0.04	75.34	±	10.80	2.70	±	0.40	0.86	±	0.03	80.70	±	10.00	Accurate
	EGCG	320.6	±	107.9	1.50	±	0.19	0.55	±	0.11	71.87	±	10.26	1.50	±	0.20	0.54	±	0.09	72.80	±	8.70	Acceptable
	EGCG3Me	10.8	±	7.4	1.44	±	0.17	0.54	±	0.11	5.08	±	0.78	1.40	±	0.10	0.47	±	0.11	5.70	±	0.70	Poor
	Total	869.7	±	423.2	2.46	±	0.46	0.83	±	0.06	171.14	±	30.96	2.50	±	0.40	0.84	±	0.05	175.70	±	28.30	Accurate
FAAs	Asp	15.4	±	10.7	2.18	±	0.31	0.80	±	0.06	5.02	±	0.62	2.20	±	0.30	0.81	±	0.05	4.80	±	0.50	Accurate
	Glu	17.6	±	8.8	1.70	±	0.18	0.66	±	0.07	5.10	±	0.54	1.70	±	0.20	0.68	±	0.07	5.20	±	0.50	Acceptable
	Arg	51.1	±	70.9	1.88	±	0.32	0.71	±	0.10	39.16	±	7.60	1.80	±	0.30	0.67	±	0.11	41.00	±	7.30	Acceptable
	Thea	69.9	±	65.3	1.98	±	0.26	0.74	±	0.07	33.52	±	4.89	2.00	±	0.30	0.74	±	0.08	34.10	±	4.70	Acceptable
	Total	181.2	±	171.7	2.25	±	0.34	0.80	±	0.06	78.23	±	11.12	2.20	±	0.30	0.78	±	0.06	81.00	±	11.00	Accurate
Caffeine		183.4	±	68.7	1.39	±	0.19	0.48	±	0.13	49.58	±	8.35	1.40	±	0.20	0.50	±	0.11	51.80	±	6.90	Acceptable

“*v*” and “*p*” in each index means validation and prediction, respectively.

^a^Prediction performance is represented as described by Chang et al., (2001); RPD*p* > 2.0, accurate prediction; 1.4 < RPD*p* < 2.0, acceptable prediction; RPD*p* < 1.4, poor prediction.

### Detection of important hyperspectral regions by DSA

**Figure 4:**
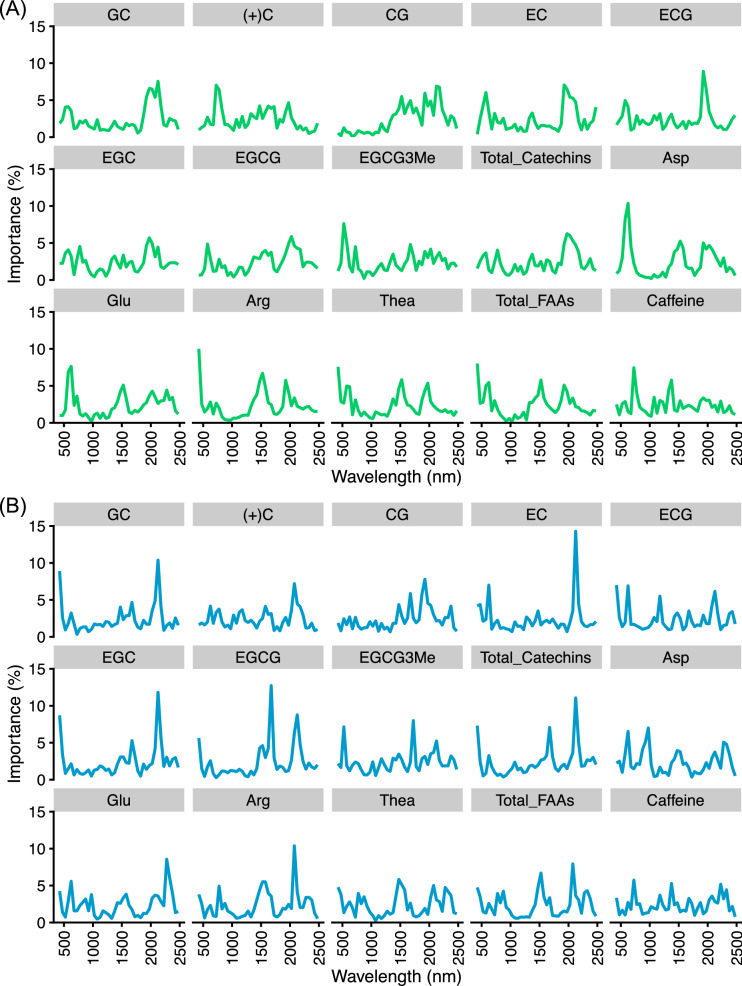
Detection of important hyperspectral regions by data-based sensitive analysis (DSA). Importance values, which were averaged over 100 replicates and accumulated at 50-nm intervals, were visualized as DSA results based on OR-Cubist (**A**) and DT-Cubist (**B**) treatment. Figures were visualized by the R package “ggplot2” ver. 3.3.2.

Data-based sensitivity analysis (DSA) was performed to detect important hyperspectral regions in models to estimate tea quality-related metabolites, and their results based on OR-Cubist and DT-Cubist were visualized at 50-nm intervals (Fig. 4). Different shapes of DSA plots were observed for caffeine and individual catechins and amino acids (Fig. 4). For catechins without CG and EGCG-3ʺMe that showed poor prediction performance, the peak region consisting of high importance values was observed around 2000 nm (Fig. 4). For amino acids, the peak region of high importance values was around 1500 nm and 2000 nm (Fig. 4), and that for caffeine was around 750 nm and 1350 nm (Fig. 4).

## Discussion

To enable the non-destructive estimation of FAAs, catechins, and caffeine as tea quality-related metabolites, we performed regression modelling by combining the VIS–NIR-SWIR (400–2500 nm) hyperspectral reflectance data and machine learning algorithms. Datasets of hyperspectral data and tea quality-related metabolite contents were obtained from approximately 200 new leaves grown under different N conditions in hydroponics or from shading cultivations. The data showed wide variation that the CV in 15 phenotypes was in the range of 33.7%–138.6% (Fig. 2). The CV of EGCG (33.7%), ECG (64.7%), EGC (66.8%), EC (78.8%), caffeine (37.4%) in this study were higher than these (EGCG, 24.2%; ECG, 24.3%; EGC, 34.7%; EC, 14.0%; caffeine, 16.7%) in the previous study^35^. These results indicate that present datasets are suitable for robust regression modelling.

We applied five pre-processing techniques (Fig. 1; FDR, CR, DT, MSC, and SNV) to the OR data to enhance the more chemically associated peaks by reducing noise from spectral data and the effects of baseline shifts and overall curvature over the OR. Then we compared the model performance in the combination of six spectral patterns (OR, FDR, CR, SNV, MSC, and DT) and five machine learning algorithms (RF, SVM, Cubist, SGB, and KELM) based on the RPD values and robustness over 100 repetitions (Supplementary Table S2). In most phenotypes, the combination of DT and Cubist (DT-Cubist) was selected most often as the best performing combination in each round among the 100 repetitions (Table 1, Supplementary Table S2). DT has been used to correct wavelength-dependent scattering effects and to account for the variation in baseline shift and curvilinearity by fitting a second-degree polynomial through each spectrum^39^. Therefore, these results suggest that pre-processing based on DT was effective in improving accuracies when VIS–NIR–SWIR (400–500 nm) hyperspectral reflectance data from plant leaves were applied to the regression modelling. Cubist algorithms can generate so-called committee models that consist of a set of consecutive rule-based models to correct the predictions of previous member models^40^; this approach is computationally efficient and well suited to big data analytics^40^. Cubist is better equipped to handle extrapolations out of range of the training target data by relying on a rule-based multivariate linear regression model rather than an ensemble of decision trees with interconnected leaves associated with rigid target predictions^41^. Furthermore, Cubist algorithms achieved the best performance in a comparison of a large collection composed of 77 popular regression models^42^. Previous studies also showed that the Cubist algorithm had the potential of an efficient model algorithm for various plant traits using reflectance data such as leaf area index^43^. Our previous study also showed that the Cubist algorithm had the best regression performance with VIS–NIR–SWIR (400–2500 nm) hyperspectral reflectance data and the contents of N and chlorophyll in tea leaves^37^. These results and previous studies strongly show that the combination of the pre-processing technique based on the DT-Cubist algorithm was suitable for regression modelling of the VIS–NIR–SWIR reflectance data in plants.

These regression models based on DT-Cubist archived that the mean RPD values in most of the 15 phenotypes were above the acceptable threshold (RPD = 1.4)^38^ except for CG and EGCG-3ʺMe (Fig. 3A). For catechins and caffeine, the mean RPD values of GC, EC, ECG, EGC, and total catechins were above the accurate threshold (RPD = 2.0)^38^, but those of EGCG and caffeine were not (Fig. 3A). A previous study based on NIR analysis of ground tea leaves indicated that the calibration models for caffeine, EGC, C, EGCG, EC, ECG, and total catechins, except for GC and EGCG-3ʺMe, had high performance with high *R*^2^ (more than 0.90)^34^. The model’s performance for EGCG and caffeine differs from that of other catechins in this study, and these may not be caused by chemical properties. In the dataset for our modelling, the CV values of EGCG and caffeine were drastically lower than those for other catechins (Fig. 2). These low variations in the reference dataset of EGCG and caffeine could have affected the regression modelling performance. Our model performance (*R*^2^ = 0.50 − 0.86) was inferior to that (*R*^2^ = 0.89 − 0.94) of the report of Huang et al.^35^ that also performed the regression modelling based on 400 − 2498 nm reflectance for some catechins and caffeine content in fresh tea new leaves. Although Huang et al.^35^ acquired the reflectance data using a near-infrared spectrometer under a dark environment in the room, we non-destructively did use a leaf clipping unit on the site under a field condition that could also cause some effect of spectral noise. These differences in measurement methods may affect the prediction performance. However, our measurement method was more designed to be applied in actual agricultural fields. In the previous work of Lee et al.^34^ and in this study, the estimation of EGCG-3ʺMe was low (Fig. 2). The EGCG-3ʺMe content in the cultivars, Benifuuki, Benifuji, and Benihomare was drastically higher than the other tea cultivars^44^, including Yabukita, which was used in this study. Adding these data for high-EGCG-3ʺMe-content cultivars to the reference data would expand the data variation and possibly improve model performance.

The contributions of hyperspectral regions to generate the regression models for tea quality-related metabolite contents were detected using DSA. The different shapes of DSA plots based on OR-Cubist and DT-Cubist were observed for caffeine and individual catechins and amino acids (Fig. 4). These results suggest that the machine learning algorithms separately determine the variable contributions of important spectral regions to estimate each metabolite. In most catechins, the peak region consisting of high importance values was observed around 2000 nm by DSA (Fig. 4). These results overlapped with spectral regions of known absorption features associated with phenolic compounds and the bending and stretching of C–H and O–H bonds^45–47^. In amino acids, the peak regions of high importance were observed around 1500 nm and 2000 nm by DSA (Fig. 4). These results were also consistent with previously reported spectral regions (e.g., 1520–1523 nm) for amino acid estimation^45^. DSA based on DT reflected the importance of these regions more than the other pre-processing patterns (Fig. 4, Supplementary Fig. S2). NIR and SWIR spectra in fresh leaf exhibit confounding factors in water absorption regions (approximately 1350–1450 and 1850–1975 nm) that may mask optical chemical features^48–50^. Our dataset also indicated that many catechins and FAAs contents were negatively and positively correlated with water content, respectively (Supplementary Figs. S3, S4). Although each metabolite in fresh tea leaves may be affected by the water content, the relationship between the model performance and the correlation of each metabolite and the water content was inconsistent (Fig. 3, Supplementary Figs. S3, S4), which indicates that the prediction model in this study has been constructed with an optimized model that takes into account the water content in fresh leaves.

The results of the present study suggest that spectroscopic analyses based on VIS–NIR–SWIR (400–2500 nm) hyperspectral reflectance data and machine learning algorithms have good potential to non-destructively estimate the contents of FAAs, catechins, and caffeine as tea quality-related metabolites in new fresh leaves (Table 2). Our modelling approaches also indicate that pre-processing techniques help to improve the accuracy of model performance. In particular, the combination of DT pre-processing methods and Cubist algorithms showed the highest model performance for most tea quality-related metabolites. These findings will contribute to the non-destructive real-time diagnosis of metabolite levels in tea cultivation management and breeding programs.

## Methods

### Plant materials

To obtain the dataset of tea quality-related metabolites contents with variations, a series of four experiments (Exp. 1 to Exp. 4) were conducted as described by Yamashita and Sonobe et al.^37^. New leaves were plucked from each experiment, and its reflectances were measured in site under a field condition. The reflectance datasets of these experiments were also used in our previous study^37^.

Exps. 1 and 2 were conducted based on hydroponic nutrient tests. One-year-old rooted tea cuttings of cv. Yabukita, a popular and leading Japanese cultivar for green tea, were used in the hydroponic cultures that were conducted under ambient light conditions in an unheated greenhouse (120 m^2^) at Shizuoka University (Shizuoka, Japan). A minor modification of the culture method described by Konishi et al. (1985) was used. Exp. 1 was conducted based on different six nitrogen (N) nutrient amount conditions using three to five biological replicates: 0 × N, 0.01 × N, 0.1 × N, 1 × N (40 mg L^−1^), 2 × N, 4 × N. After approximately 6 months of treatment, one or two new leaves were plucked from one individual. Exp. 2 was conducted based on low-light conditions (85% shading) and different four N nutrient amount conditions using three biological replicates: 0 × N, 0.1 × N, 1 × N, 4 × N. After 23 days for treatment, one or two new leaves were plucked from one individual.

Exp. 3 was conducted using mature tea plants (ridges) of cv. Yabukita at Shizuoka University (Shizuoka, Japan) based on low-light conditions (85% shading). New leaves in each leaf-stage were plucked from approximately random 15 shoots in sunlight and shaded tea ridges, and a total 87 leaves In Exp. 4, new leaves in each leaf-stage were plucked from approximately 20 shoots in a 7-year-old rooted tea cutting of a Japanese albino cultivar cv. Koganemidori, which had been bred from the natural etiolated bud sport, in hydroponics.

Finally, 215, 201, and 201 leaves samples in each experiment were freeze-dried, grounded into a fine powder, and then analyzed for free amino acids (FAAs), catechins, and caffeine, respectively.

### Reflectance measurements and pre-processing

Reflectance data in new leaves was measured by an ASD FieldSpec4 unit (Analytical Spectral Devices, Boulder, CO, USA) with a leaf clipping (diameter 20 mm) (Supplementary Fig. S5). The widest part in the center of the leaf was measured three times so that a leaf clipping could fit inside the leaf and the average value of that was taken as the representative for each leaf. This spectroscopy contained three detectors, visible (VIS) and near-infrared (NIR), short-wave infrared (SWIR), and SWIR 2. ViewSpec Pro Software (Analytical Spectral Devices) was used to correct differences in the spectral drifts at 1000 and 1800 nm caused by inherent variation in these detector sensitivities. Finally, OR data were recorded with a sampling resolution of 1 nm steps across the entire wavelength domain from 400 to 2500 nm. Five pre-processing methods were also tested based on their success in previous studies, namely first FDR, CR, SNV, MSC, and DT. FDR is effective in reducing baseline variation and increasing the resolution of spectral peak features^51,52^. CR is a brightness normalization technique that has been applied to enhance related changes^53^. MSC and SNV have also been used to eliminate the effect of noise, baseline drift, and light scattering of the spectrogram^54–56^. DT has been used to correct wavelength-dependent scattering effects and accounts for the variation in baseline shift and curvilinearity by fitting a second-degree polynomial through each spectrum^39^. All methods were performed using R version 3.6.3 and the R package “prospectr” ver. 0.2.0.

### Measurement of tea quality-related metabolites

Catechins and caffeine contents were measured according to the methods described by Horie et al.^57^ and Yamashita et al.^58^. Dry ground leaf tissue (25 mg) was added to 5 mL of 50% (v/v) acetonitrile and shook with 130 strokes min^−1^ for 60 min at room temperature. The suspended samples were centrifuged at 2000×*g* for 15 min at 4 °C, and then the supernatants were individually passed through 0.45-µm polytetrafluoroethylene filters (Advantec, Tokyo, Japan). The resulting solutions were stored at − 30 °C until they were analyzed by HPLC as described by Yamashita and Uchida et al.^58^. The eight catechins, GC, C, CG, EC, ECG, EGC, EGCG, EGCG-3ʺMe, and caffeine were quantified. Their total value without caffeine was also expressed as total catechins.

The FAAs contents were measured according to the method described by Goto et al.^59^ and Yamashita et al.^58^. Dry ground leaf tissue (10 mg) was added to 10 mg of polyvinylpolypyrrolidone and 5 mL of ultra-pure water and was shook with 130 strokes min^−1^ for 60 min at room temperature. The suspended samples were centrifuged at 2000×*g* for 15 min at 4 °C, and then the supernatants were individually passed through 0.45-µm cellulose acetate filters (Advantec). The resulting solution was stored at − 30 °C until analysis by HPLC as described by^58^. Nine amino acids [Asp, asparagine (Asn), Glu, glutamine (Gln), serine (Ser), Arg, alanine (Aln), Thea, and γ-aminobutyric acid (GABA)] were quantified. Their total value was also expressed as total FAAs.

### Regression models based on machine learning algorithms

The regression modelling was conducted as described by Yamashita and Sonobe et al.^37^ with minor modification and its flow chart was shown in Supplementary Fig. S1. For modelling, a stratified random sampling approach was applied, for which strata were formed based on experiments and treatments, and then all measurements were divided into three dataset groups as follows; a training set (50%), which was used to fit the models; a validation set (25%), which was used to estimate the prediction error for model selection; and a test set (25%), which was used for assessing the generalization error in the final selected model. To evaluate the robustness of models, this flow was repeated 100 times before pre-processing the OR and generating regression models.

When performing regression modelling based on machine learning algorithms, a genetic algorithm (GA)-based approach was applied to select wavelengths using the “ga_pls” function (with the parameter “GA.threshold” and others set as 50 and default values, respectively) of the R package “plsVarSel” ver. 0.9.6. and R ver. 3.6.3. GA were effective for removing noninformative wavelengths to construct simpler and better prediction models. Regression models were then constructed from the selected wavelengths using the following representative five algorithms: RF, SVM, Cubist, SGB, and KELM. The overviews of these five algorithms were described in Supplementary Table S1.

RF was performed and optimized with the five hyperparameters by the R package “randomForestSRC” ver. 2.9.3. SVM was performed with the Gaussian radial basis function kernel and optimized with the two hyperparameters by the R package “e1071” ver. 1.5-8. Cubist was performed and optimized with the two hyperparameters by the R package “Cubist” ver. 0.2.3. SGB was performed and optimized with the four hyperparameters by the R package “gbm” ver. 2.1.5. KELM was performed and optimized with the two hyperparameters by the MATLAB and Statistics Toolbox Release 2016a (MathWorks, Natick, MA, USA; source code downloaded from https://www.ntu.edu.sg/home/egbhuang/). The optimizations in the hyperparameters of these machine learning algorithms were conducted based on the Bayesian optimization approach that was applied with the Gaussian process^60,61^ using the R package “rBayesianOptimization” ver. 1.1.0. The hyperparameters information of these algorithms is shown in Supplementary Table S1.

The validation (*v*) and prediction (*p*) accuracy of constructed models was assessed based on the following three indexes: the ratio of performance to deviation (RPD), the coefficient of determination (R^2^), root-mean-square error (RMSE). The performance of the prediction model was assessed according to the following three classes of RPD^38,62,63^: RPD > 2, accurate prediction; RPD of 1.4–2, acceptable prediction; RPD < 1.4, poor prediction.

### Data-based sensitivity analysis (DSA)

To extract human-understandable knowledge from supervised learning black box data mining models, we performed the DSA^64,65^ by using the “Importance” function of the R package “rminer” ver. 1.4.5, as previously described by Yamashita and Sonobe et al.^37^. Although DSA is similar to a computationally efficient one-dimensional sensitivity analysis^64^, this method uses several training samples instead of a baseline vector^65^ and it could be applied to black-box functions by querying the fitted models with sensitivity samples and recording their responses.

## Supplementary Information

Supplementary Table S1.

Supplementary Table S2.

Supplementary Figures.
