# Supplementary material for: Potential of spectroscopic analyses for non-destructive estimation of tea quality-related metabolites in fresh new leaves
Source: Sci Rep. 2021 Feb 18;11:4169. doi: 10.1038/s41598-021-83847-0 (PMC7892543; doi:10.1038/s41598-021-83847-0)
Supplement: Supplementary file 3 — Supplementary Figures. [file 41598_2021_83847_MOESM3_ESM.pdf]

Supplementary Information

Potential of spectroscopic analyses for non-destructive estimation of tea quality-related metabolites in fresh new leaves

Hiroto Yamashita[1,2], Rei Sonobe[1,3]*, Yuhei Hirono[3,4], Akio Morita[1,3] and Takashi Ikka[1,3]*

*Corresponding authors
Rei Sonobe (sonobe.rei@shizuoka.ac.jp)
Takashi Ikka (ikka.takashi@shizuoka.ac.jp)

<u>This PDF file includes:</u>
Supplementary Figure S1 Flow charts of the regression modelling by machine learning algorithms.

Supplementary Figure S2 Detection of important hyperspectral regions by data-based sensitive analysis (DSA) in each pre-processing spectral.

Supplementary Fig. S3 Correlation matrix of catechins, caffeine contents, and other chemical properties.

Supplementary Fig. S4 Correlation matrix of free amino acids content and other chemical properties.

Supplementary Fig. S5 Field-portable spectroradiometers (ASD FieldSpec4 unit, Analytical Spectral Devices, Boulder, CO, USA) with a leaf clipping (diameter 20 mm) that used in this study (A). Representative tea leaves for measurement (B). Bar = 3 cm.

<u>Supplementary Data file includes:</u>
Supplementary Table S1. Tuning hyperparameters in each machine learning algorithm
Supplementary Table S2. Model performance of all combinations of the six pre-processing patterns and five machine learning algorithms in each round.

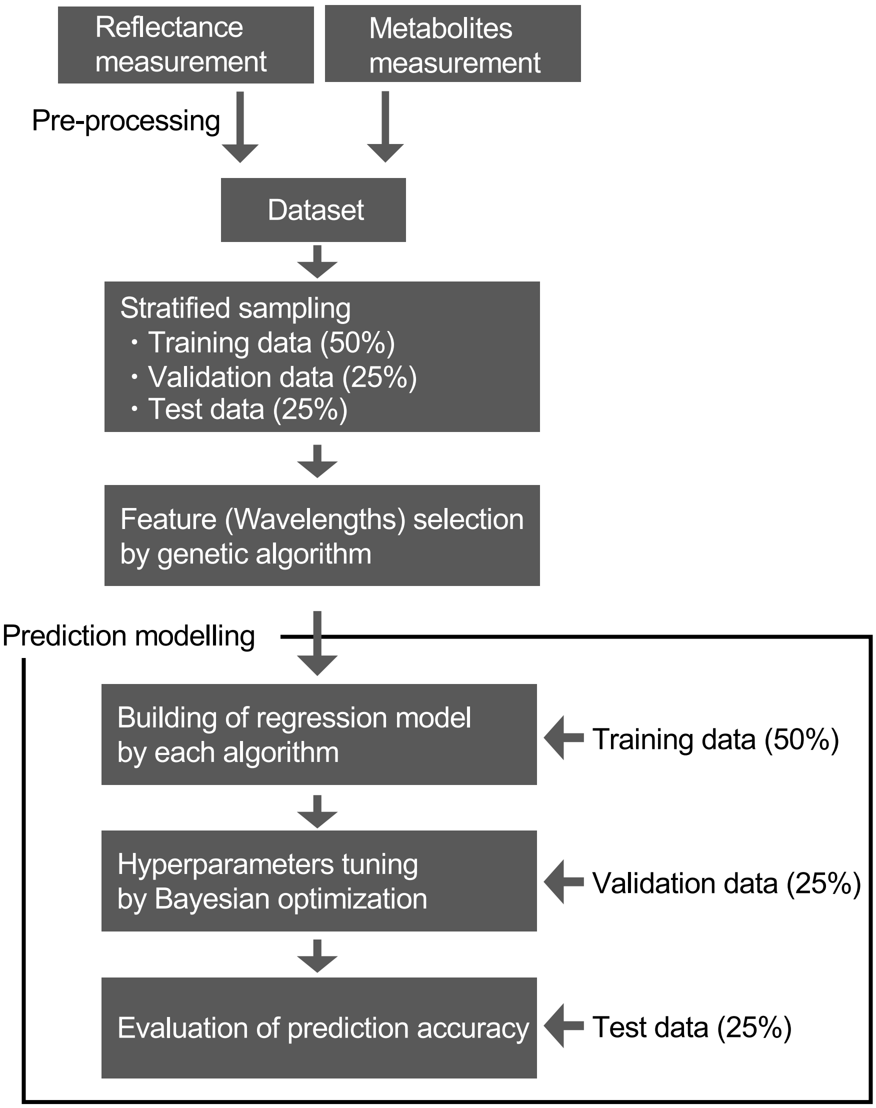

Supplementary Figure S1 Flow charts of the regression modelling by machine learning algorithms

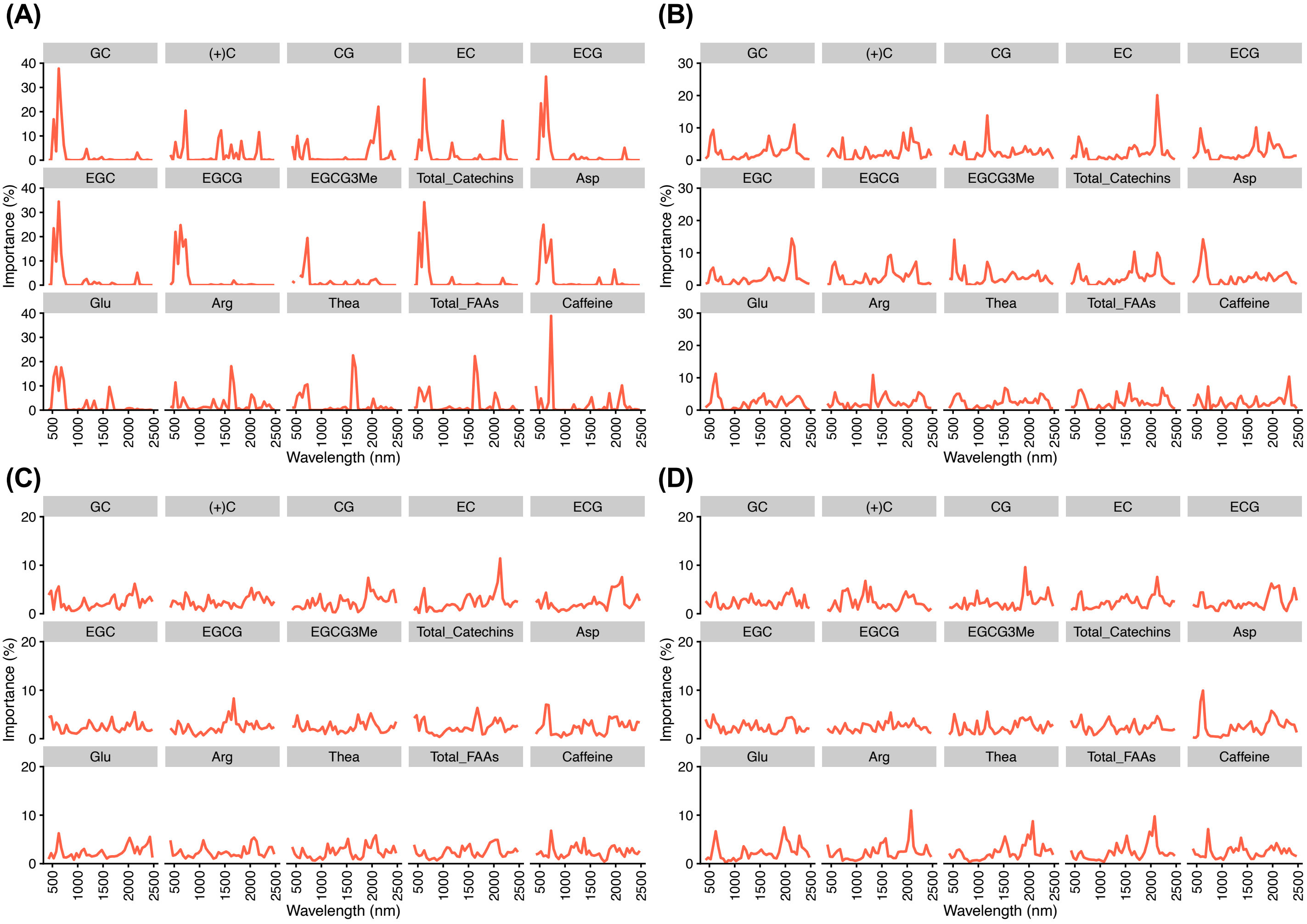

Supplementary Figure S2 Detection of important hyperspectral regions by data-based sensitive analysis (DSA) in each pre-processing spectral.
Importance values, which were averaged over 100 replicates and accumulated at 50-nm intervals, were visualized as DSA results based on FDR-Cubist (A), CR-Cubist (B), MSC-Cubist (C), and SNV-Cubist (D) treatment. Figures were visualized by the R package “ggplot2” ver. 3.3.2.

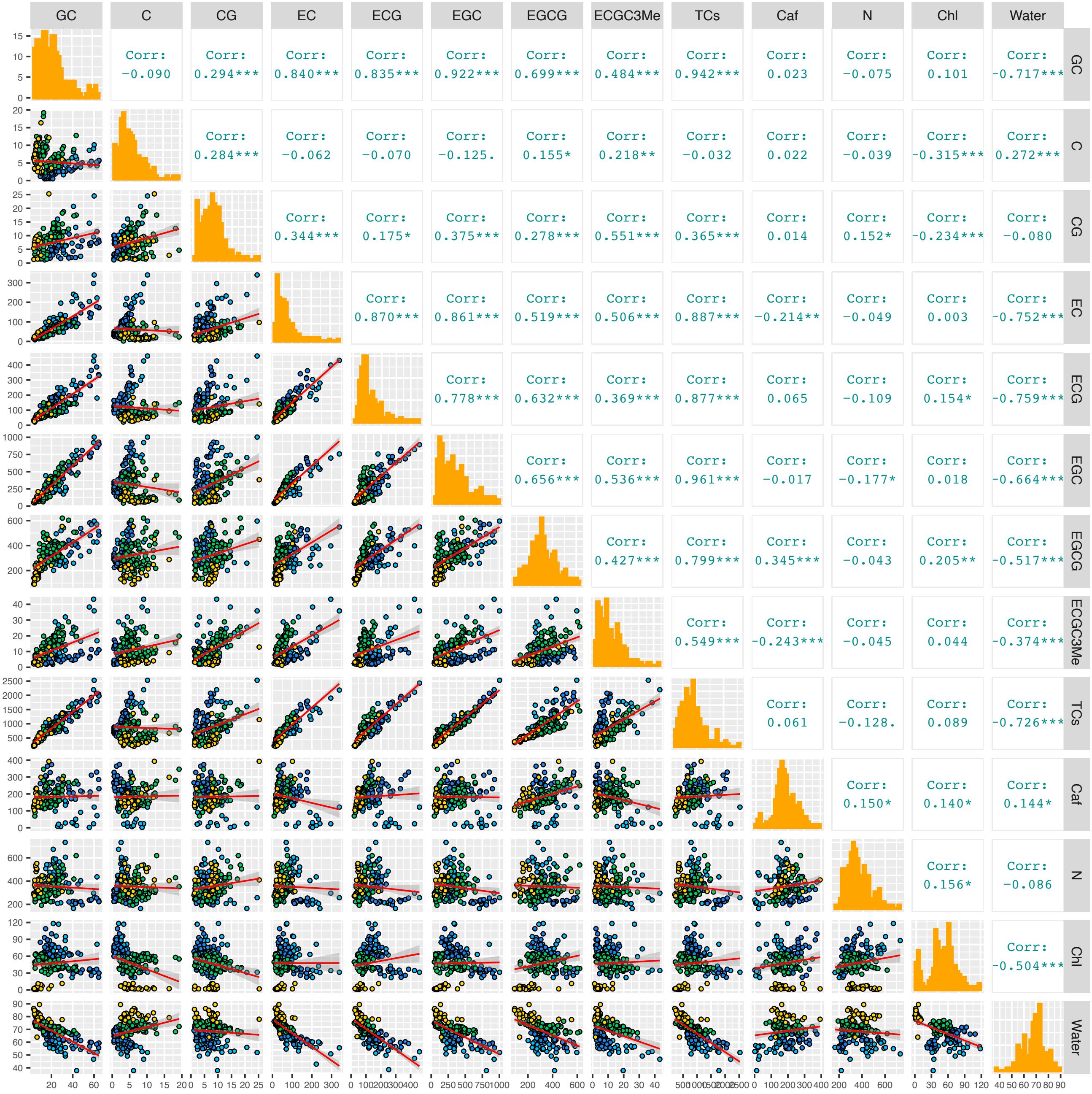

Supplementary Figure S3 Correlation matrix of catechins, caffeine contents, and other chemical properties.

Data for nitrogen, chlorophyll, and water contents were used from our previous study (Yamashita, Sonobe, et al., 2020). Correlation matrix was visualized by the R packages “ggplot2” ver. 3.3.2 and “GGally” ver. 2.0.0.

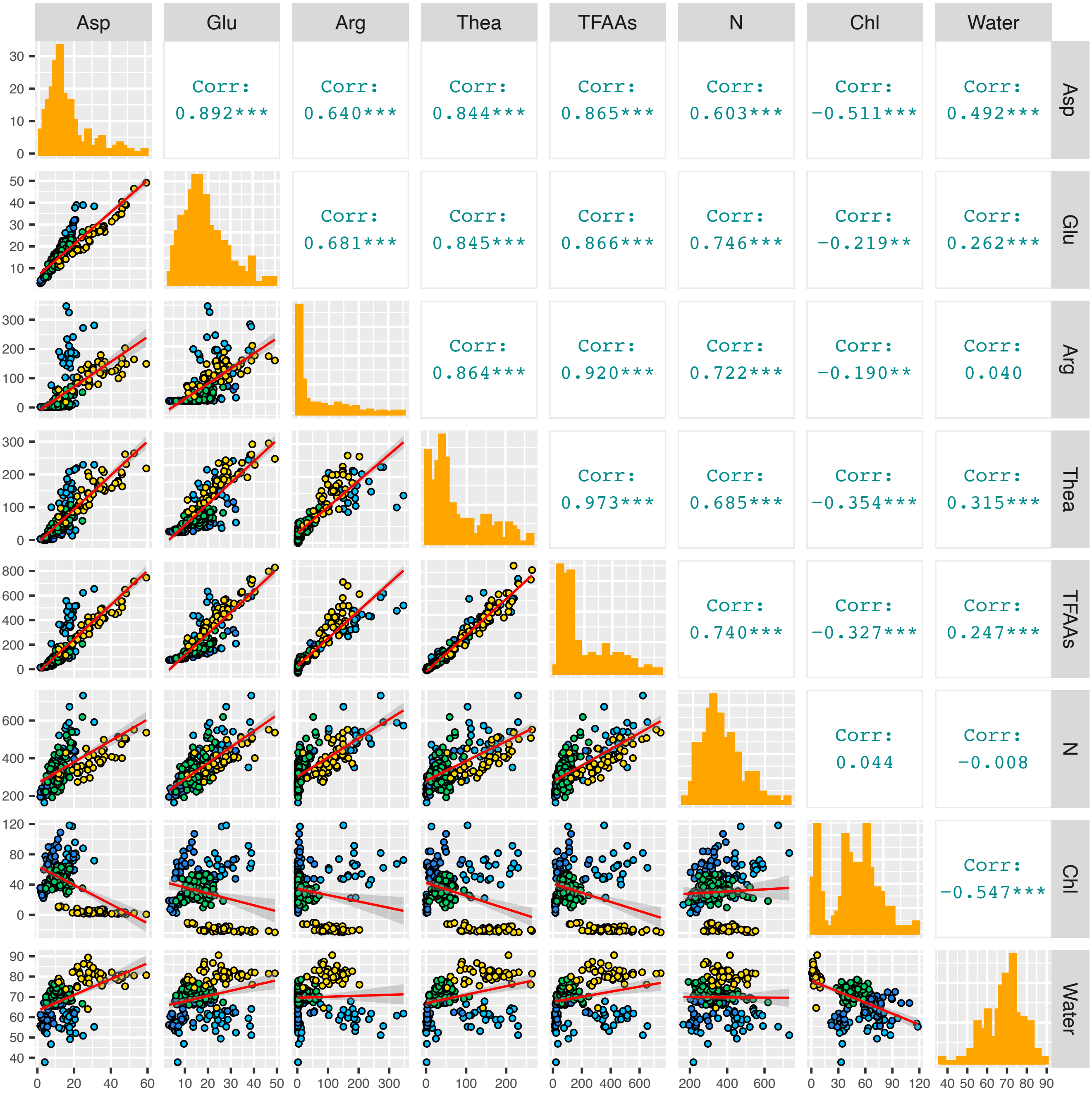

Supplementary Figure S4 Correlation matrix of free amino acids content and other chemical properties.

Data for nitrogen, chlorophyll, and water contents were used from our previous study (Yamashita, Sonobe, et al., 2020). Correlation matrix was visualized by the R packages “ggplot2” ver. 3.3.2 and “GGally” ver. 2.0.0.

(A)

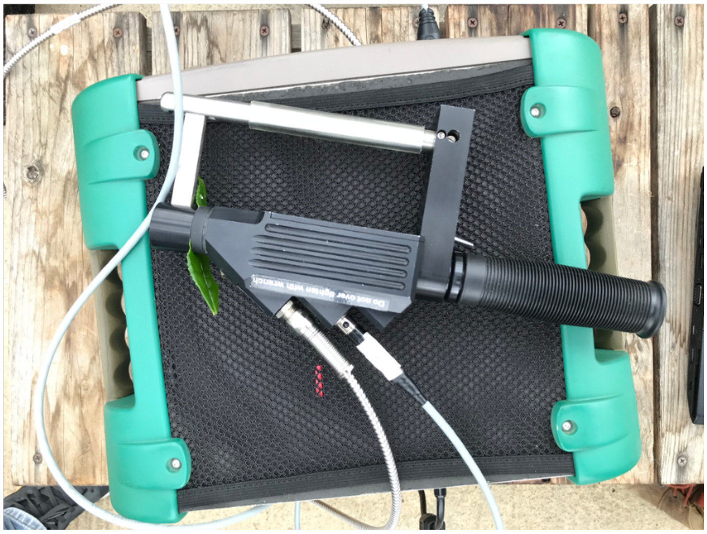

(B)

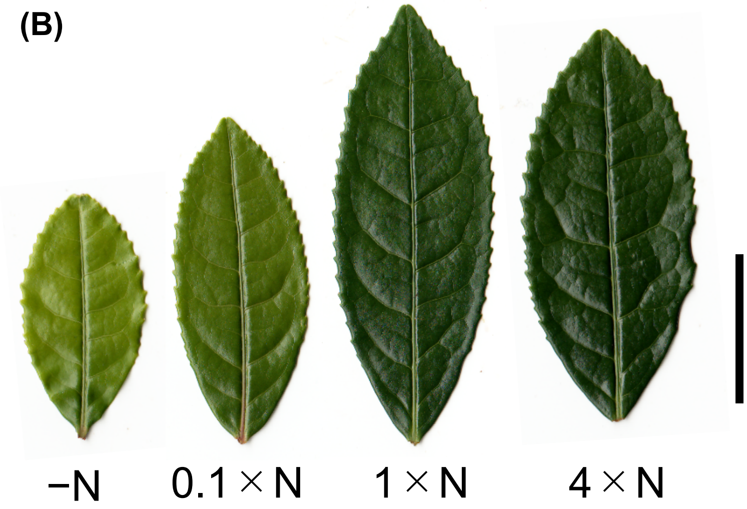

Supplementary Figure S5 **Field-portable spectroradiometers (ASD FieldSpec4 unit, Analytical Spectral Devices, Boulder, CO, USA) with a leaf clipping (diameter 20 mm) that used in this study (A). Representative tea leaves for measurement (B). Bar = 3 cm.**
